# Meta-analysis of the effects of the dietary inclusion of brewers grains on feed intake, milk yield, and feed efficiency of lactating dairy cows

**DOI:** 10.3168/jdsc.2024-0626

**Published:** 2024-12-12

**Authors:** S.C. Chelkapally, T.H. Terrill, I.M. Ogunade, Z.M. Estrada-Reyes, A.A. Pech-Cervantes

**Affiliations:** 1 Agricultural Research Station, Fort Valley State University, Fort Valley, GA 31030; 2 Division of Animal and Nutritional Science, West Virginia University, Morgantown, WV 26505; 3 Department of Animal Science, North Carolina A&T State University, Greensboro, NC 27411; 4 College of Agriculture, Food and Natural Resources, Cooperative Agricultural Research Center, Prairie View A&M University, Prairie View, TX 77446

## Abstract

Distillery byproducts such as brewers grains (BG) have been widely used in dairy cow diets for decades to reduce diet costs and improve nutrient efficiency. Thus, the objective of this study was to evaluate the magnitude of the effects of dietary supplementation with BG on feed intake (DMI), digestibility, milk yield (MY), and feed efficiency (FE) of lactating dairy cows. A total of 12 peer-reviewed articles (n = 472 dairy cows) from 1983 to 2021 were collected systematically with the PRISMA method. The weighted raw mean differences (RMD) between dietary BG and control treatments were estimated with a robust variance estimation. Likewise, diet characteristics, such as CP content, NDF content, type of BG (wet = 1 and dry = 2), DIM, and BG inclusion rate (0 to 75%) were used as covariates in a meta-regression, subset, and dose-response analysis. Compared with the control, dietary BG decreased DMI (19.7 vs. 20.2 kg/d) and tended to increase MY (28.9 vs. 28.4 kg/d), but no effects were observed on FCM yield (29.1 vs. 28.8 kg/d), milk fat (3.8 vs. 3.7%), and milk protein (3.2 vs. 3.2%) concentrations. However, dietary BG increased FE (1.45 vs. 1.34 FCM/DMI) without influencing total-tract DM (61.9 vs. 55.7%) and NDF (49.7 vs. 54.5%) digestibility. Increasing the inclusion rate of BG linearly increased (R^2^ = 0. 554) dietary NDF. Thus, the dose-response analysis revealed that feeding up to 20% BG (dietary NDF = 39%) to lactating dairy cows increased MY and FE. Overall, this meta-analysis supports the hypothesis that dietary BG improved FE in lactating dairy cows.

## Highlights

• Dietary BG decreased DMI but increased feed efficiency in lactating dairy cows.
• Dietary BG should not exceed 20% in the diet of lactating dairy cows.
• Dietary BG linearly increases neutral detergent fiber content in the diet across studies.

## Graphical Abstract

**Figure:**
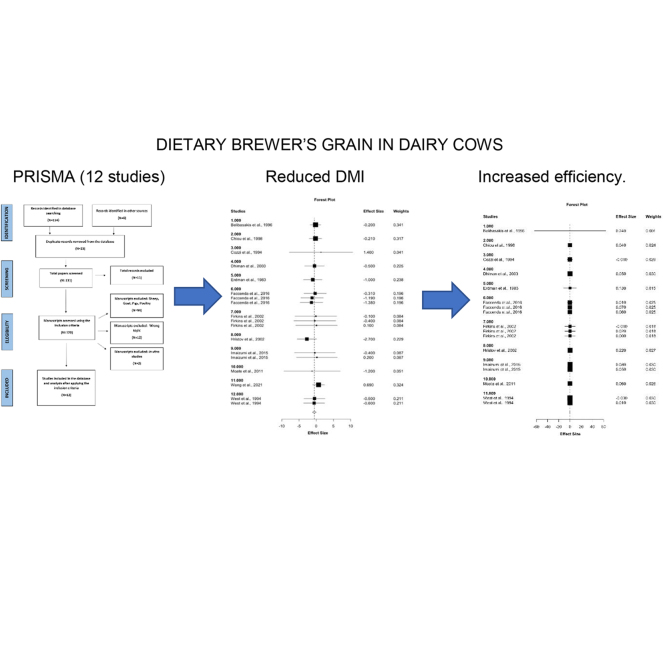
**Summary:** Dietary inclusion of brewers grains (BG) in dairy cows decreased dry matter intake (DMI) but increased feed efficiency across the literature.

Brewers grain (**BG**), or brewery waste, is one of the most important industrial wastes produced by the brewing industry and collected during the manufacturing of cereal malt beverages (Chanie and Fievez, 2017; Naik et al., 2018). Feeding byproducts such as BG could help to reduce waste accumulation and environmental pollution, as well as reduce costs without compromising the nutritional needs of dairy cows (Souza et al., 2016; Faccenda et al., 2017). Traditionally, dried grains have a wide range of purposes, such as the production of fertilizer for increasing agricultural productivity, energy production, and animal feed (Mussatto et al., 2006). According to Dhiman et al. (2003), the residue can be marketed directly as dried brewers grains or wet BG. Both products are extracted barley malt residue produced during the wort, or beer-making process, alone or in combination with other cereal grains or grain products (Murdock et al., 1981; Chiou et al., 1998; Moate et al., 2011). Every 100 (378.5 L) gallons of beer produced yields about 20 kg of BG. The total beer production in 2015 was 1.37 billion hectoliters and a total of 36 billion of BG (Olajire, 2021). Thus, BG production is substantial around the world. Therefore, more efforts should be made to effectively use BG, particularly in developing countries where BG utilization is currently lower (Mussatto et al., 2006). As a feedstuff, BG has approximately 28.4% CP, 47.1% NDF, and 5.2% ether extract on a DM basis (NRC, 2001; Souza et al., 2016). Brewers grains are an excellent alternative for ruminants, particularly dairy cows, to balance the high starch content of their diets because of their low energy content and fibrous nature (Dhiman et al., 2003). Furthermore, BG is rich in essential and nonessential AA, vitamins, and important mineral components (Lao, 2021). Lysine and methionine are the first AA to limit milk yield (**MY**) in dairy cows. Although BG were substituted for soybean meal in dairy feeds because of the high protein content and healthy, balanced AA profile, RUP from BG is an excellent source of protein because it contains high levels of lysine and methionine (Cozzi and Polan, 1994; Belibasakis and Tsirgogianni, 1996).

Brewers grains, as a source of fiber, protein, and potentially beneficial phenolic compounds such as ferulic, p-coumaric, and caffeic acids, are gaining demand due to their antioxidant properties and diverse range of potential bioactivities (Ikram et al., 2017). Previous research showed that dairy cattle fed with high amounts of BG had higher rumen acetic acid concentrations, DMI, and milk fat percentage (Davis et al., 1983). Comparably, a small number of studies discovered notable variations in MY and fat content, but not in protein or milk fat, in dairy cows fed with BG (Polan et al., 1985; Dhiman et al., 2003; Hristov and Ropp, 2003). Therefore, the optimal amount of BG inclusion in dairy cattle diets is still unknown, even though many researchers examined the effect of BG on cattle performance (Firkins et al., 2002; Faccenda et al., 2017). Thus, we hypothesize that using a meta-analytic approach will be a suitable research strategy to estimate the optimum inclusion rate of BG in dairy diets. Therefore, the objective of this study was to evaluate the effects of dietary supplementation with BG on feed intake, milk production, and feed efficiency (**FE**) of lactating dairy cows following a meta-analytic approach.

This study used previously reported data across the literature, and it did not require the use of animals. Thus, no Institutional Animal Care and Use Committee protocol was required. To create a comprehensive database, a systematic search using the scientific databases ScienceDirect, Google Scholar, PubMed, The Web of Science, Scopus, and the Directory of Open Access Journals, following the methodology outlined by Oliveira et al. (2017) and Arriola et al. (2021), was conducted. The following key words: “dairy cattle,” “intake,” “BG,” “performance,” “milk production,” and “feed efficiency,” were used. The data extraction process followed the PRISMA approach of Moher et al. (2009). A total of 154 peer-reviewed publications were found in the database, but 119 were excluded from the analyses for the following reasons: 11 of the articles did not have sufficient data analysis and reporting, 12 had the incorrect topic, 94 had the incorrect livestock species, and 2 were in vitro research studies. Following the inclusion criteria, 12 articles (Table 1) with 472 dairy cattle (578.9 ± 76.69 kg; ± SD) were assigned to 19 treatment comparisons, with BG inclusion rates in diets ranging from 0% to 75%.

**Table 1:** List of studies included in the meta-analysis of the effects of dietary inclusion of brewers grains (BG) on feed intake, milk production, and feed efficiency of lactating dairy cows

Study	N	DIM		Type of BG
Belibasakis and Tsirgogianni, 1996	20	60		Wet
Chiou et al., 1998	32	21		Wet
Cozzi and Polan, 1994	24	22		Dry
Dhiman et al., 2003	24	56		Both
Erdman and Vandersall, 1983	24	28		Dry
Faccenda et al., 2017	5	88		Dry
Firkins et al., 2002	60	70		Wet
Hristov and Ropp, 2003	4	323		Dry
Imaizumi et al., 2015	40	168		Wet
Moate et al., 2011	16	200		Wet
Wang et al., 2021	42	210		Wet
West et al., 1994	20	145		Wet

The weighted raw mean differences (**RMD**) between the control and BG treatments were used to determine the overall effect size with the following formula: RMD = $\frac{\sum W_{i}\cdot \theta_{i}\;}{\sum W_{i}},$ where *W_i_* = weight for the study *i* and *θ_i_* = effect size from study *i*. Following Tipton (2015), the computed weighting made use of the inverse of variance in a hierarchical effects model with a robust variance estimation. Likewise, Higgins' (Higgins, 2008) proposed *I*^2^, which is the ratio of the treatment's variance effects to the overall variance observed, was used to determine the heterogeneity (Lean et al., 2018). Furthermore, the previously stated methods of Hedges et al. (2010) and Fisher and Tipton (2015) were used to determine the variance component between clusters (*τ*^2^) and between-studies-within-cluster (Ω^2^). Briefly, the reported SEM was used to determine the pooled SD for each comparison in the study (BG vs. control) using the formula SD = SEM × √n, where n is the number of experimental units. After that, the weighted RMD (SEM and n), *τ*^2^, and Ω^2^ statistics were taken into consideration for calculating the total effect size. Furthermore, the methodologies outlined by Egger et al. (1997) and implemented by Arriola et al. (2021) were used to quantify publication bias. As used by Pech-Cervantes et al. (2022), standardized residuals less than 2.3 were considered acceptable after outliers and significant (*P* < 0.05) points were eliminated using Cook's distance calculations. To prevent overweighting of the studies with low SEM (crossover studies), trimming was applied using the method described by Arriola et al. (2021). To determine which covariates (CP, NDF, DIM), and if type (dry = 1 and wet = 2) and inclusion level in the diet of BG had an influence on the effect size in the response variables, a meta-regression analysis was done as follows: RMD = *S_i_* + *β*_0_ + *β*_1_·NDF + *β*_2_·CP + *β*_3_·DIM + *β*_4_·type + *β*_5_·dose + *ϵ*, where *S_i_* = study with n comparisons, *β* are coefficients corresponding to the predictors, and *ϵ* is the error term. Following the steps outlined by Viechtbauer (2010) and Oliveira et al. (2017), the Wald test multiparameter technique was used to determine the effect of the covariates on the model. The dose-response and trend were computed using the Greenland (Greenland and Longnecker, 1992; Greenland, 1995) method, modified by Pech-Cervantes et al. (2022), when the variables were significant (*P* < 0.05). Using funnel plots and Egger's regression approach between RMD and SE, publication bias has been calculated and illustrated (Egger et al., 1997; Oliveira et al., 2017). Similarly, using the technique outlined by Arriola et al. (2021), Cook's distances were used to exclude outliers and influential points. The robumeta (version 1.3.1093; https://cran.r-project.org/web/packages/robumeta/robumeta.pdf) and metafor (version 1.3.1093; https://cran.r-project.org/web/packages/metafor) packages in Rstudio (version 1.25), following Fisher et al. (2015) and Viechtbauer (2010), were used for all data analyses, including RMD, forest plot, and meta-regression analysis.

The chemical composition remained consistent throughout the literature despite the high heterogeneity within studies (*P* > 0.05), and no influential points were detected. However, increasing the inclusion of BG in the diet linearly increased NDF content (R^2^ = 0.62). When compared with the control, dietary BG did not affect (*P* > 0.05) the total-tract digestibility of DM (61.95% vs. 55.75%), CP (64.5% vs. 61.67%), or NDF (49.84% vs. 54.74%). Likewise, there was no difference (*P* > 0.05) between the control and the BG-supplemented diets on milk fat (1.08 vs. 1.07 kg/d) and milk protein yield (0.94 vs. 0.96 kg/d). Moreover, feeding BG did not differ from the control in terms of milk fat, milk protein, or milk lactose concentration (%). However, among treatment comparisons, the dietary inclusion of BG decreased (*P* = 0.05) DMI (20.25 vs. 19.71 kg/d) compared with the control (Figure 1a). Conversely, dietary BG supplementation tended (*P* = 0.1) to increase MY (28.46 vs. 28.93 kg/d) across treatment comparisons and was associated with greater (*P* < 0.05) FE among treatment (1.40 vs. 1.45 FCM/DMI) comparisons (Figure 1b).

**Figure 1:**
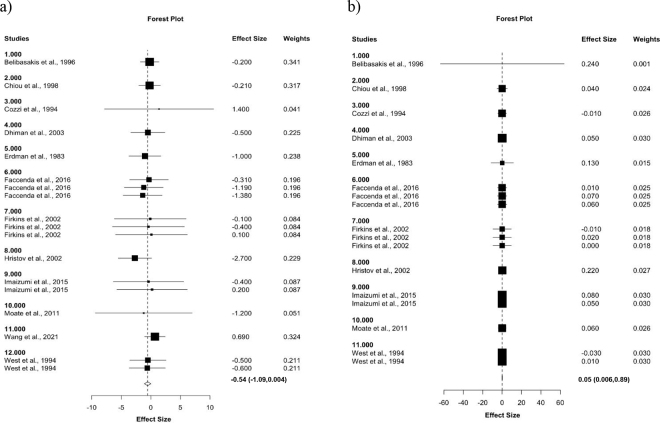
Forest plot of the effect of dietary inclusion of brewers grains (BG) on (a) DMI and (b) feed efficiency in lactating dairy cows. The x-axis shows the RMD between BG and control; squares on the left indicate a decrease in DMI, whereas squares on the right indicate an increase in DMI by BG. Lines connected to the squares correspond to 95% CI. The dotted vertical line represents the overall size effect estimate, and the diamond at the bottom represents the mean response across the studies. References: Belibasakis et al., 1996, corresponds to Belibasakis and Tsirgogianni, 1996; Chiou et al., 1998; Cozzi et al., 1994, corresponds to Cozzi and Polan, 1994; Dhiman et al., 2003; Erdman et al., 1983, corresponds to Erdman and Vandersall, 1983; Faccenda et al., 2017; Firkins et al., 2002; Hristov et al., 2003, corresponds to Hristov and Ropp, 2003; Imaizumi et al., 2015; Moate et al., 2011; Wang et al., 2021; West et al., 1994.

The variance component analysis showed a larger Ω^2^ for FE across comparisons between studies and a lower Ω^2^ for DMI, MY, and milk components. The results of the funnel test showed a low heterogeneity (*I^2^*) for the following parameters: MY, milk fat (kg/d), milk protein (%), digestibility of DM, digestibility of CP, and NDF (Table 2). Furthermore, there was only moderate heterogeneity in DMI, FCM, milk fat (kg/d), and milk protein (kg/d), but there was higher heterogeneity in milk lactose (*I^2^* > 70%). Collectively, the heterogeneity analysis (*P* > 0.05) indicated that there was not a significant publication bias among the comparisons. Both linear and categorical covariates did not affect the intercepts (*P* > 0.05) in the meta-regression. However, they decreased the Ω^2^ for all response variables except FE. Moreover, across treatment comparisons, the NDF content in the diet strongly influenced (*P* < 0.01) the responses observed in milk protein yield (cubic response R^2^ = 0.61) but did not affect the other response variables. The subset analysis revealed that wet BG tended (*P* = 0.1) to decrease DMI (20.1 vs. 20.6 [−1.09, 0.13 kg/d]) compared with the control, but no effects were observed in dried BG (18.2 vs. 19.1 [−6.8, 5.1 kg/d]). Similarly, FE was increased by wet BG (1.44 vs. 1.41 [−0.01, 0.07 FCM/DMI]) compared with the control, but no effects were observed in dried BG (1.35 vs. 1.37 [−0.2, 0.4 FCM/DMI]). However, MY (dried = 28.2 vs. 27.7 [−2.38, 3.3 kg/d]; wet = 29.1 vs. 29.8 [−0.9, 1.5 kg/d]), FCM (dried = 28.2 vs. 27.9 [−2.9, 3.5 kg/d]; wet = 29.4 vs. 29.3 [−2.3, 2.3 kg/d]) were similar between wet and dried BG with respect to the control.

**Table 2:** Effect of dietary supplementation of brewers grains (BG) on intake (DMI), milk production (MY), and feed efficiency (FE) of lactating dairy cows^1^

Item^5^	Control^2^			RMD^3^		Variance component^4^		Bias	
	N^6^	Mean	SD	Effect size	*P*-value	Ω^2^	*τ*^2^	Funnel test^7^ (*P-*value)	*I^2^* (%)
DMI (kg/d)	18	20.25	3.21	−0.54(−1.09,0.004)	0.05	2.29	0	0.08	27.47
DMD (%)	4	61.95	2.32	−6.24 (−30, 17.5)	0.18	0	0	0.21	0
CPD (%)	4	64.5	9.43	−2.83 (−21.5, 15.8)	0.30	0	0	0.34	0
NDFD (%)	4	49.65	8.54	4.89 (−6.87, 16.7)	0.12	0	0	0.85	0
MY (kg/d)	18	28.46	8.24	0.47 (−0.14, 1.09)	0.10	2.21	0	0.62	0
FCM (kg/d)	16	28.84	7.70	0.25 (−0.65, 1.16)	0.50	3.94	0	0.98	25.07
Milk fat (%)	18	3.81	0.63	−0.07 (−0.22, 0.06)	0.25	1.95	0	0.21	42.35
Milk fat (kg/d)	16	1.08	0.20	0.005 (−0.03, 0.04)	0.71	0.1	0	0.33	0
Milk protein (%)	18	3.23	0.31	−0.03 (−0.08, 0.02)	0.20	0.83	0	0.37	0
Milk protein (kg/d)	16	0.94	0.20	0.009 (−0.02, 0.04)	0.43	0.07	0	0.92	50.17
Milk lactose (%)	11	4.63	0.18	0.07 (−0.07, 0.22)	0.25	1.25	0	0.47	71.93
FE (FCM/DMI)	17	1.40	0.22	0.05 (0.006, 0.89)	0.03	33.64	0	0.76	0

1 Positive values in raw mean difference (RMD) indicate an increase by the addition of BG, whereas negative values in RMD indicate a decrease by the addition of BG with respect to the control.

2 No BG in the diet.

3 Raw mean difference between control versus BG treatment.

4 Ω^2^ = between-studies-within-cluster variance component; *τ*^2^ = between-cluster variance component (Hedges et al., 2010; Fisher and Tipton, 2015).

5 DMD = dry matter digestibility; CPD = crude protein digestibility; NDFD = neutral detergent fiber digestibility.

6 Total number of comparisons (BG vs. control).

7 *P*-value for χ*^2^* (*Q*) test for heterogeneity; *I^2^* = proportion of total variation of size effect estimated due to heterogeneity.

Results of the meta-regression analysis suggest that wet BG increased influenced RMD compared with dried BG. However, FE, milk production, and feed intake characteristics were not influenced by the CP content in diet across treatment comparisons. Similarly, the lactation status expressed as DIM did not affect the magnitude of effects (RMD) among treatment comparisons. Feed efficiency, milk components, and feed intake responses were not directly influenced when incorporating BG into the diet. However, the dose-response analysis revealed that dietary NDF influenced MY (R^2^ = 0.38) and milk protein yield (R^2^ = 0.62). In contrast, the dose-response analysis showed that dietary NDF had a low influence on FE compared with DIM (R^2^ = 0.25 vs. 0.32).

Several factors, such as temperature, fermentation process, milling procedure, and grain quality, can influence the quality and chemical composition of BG (Lao, 2021). Thus, the results of this meta-analysis showed that the type (wet or dried) and dietary inclusion level of BG influenced the magnitude of effects among treatment comparisons. The differences observed between dried and wet BG could be explained partially by the composition of BG, the type of hop used, harvest time, malting and mashing procedures, and adjuncts added or removed throughout the brewing process (Nyhan et al., 2023). Even yet, the current meta-analysis indicates that the animal responses were consistent across studies, as these variations did not increase the publication bias (*I* value).

In agreement with previous research, the covariates included in this meta-analysis partially explained the variation associated with milk production, milk composition, and FE (West et al., 1994; Dhiman et al., 2003). Although BG can alter the fiber composition in dairy cow diets, the relationship between the dietary inclusion of BG in the diet and animal performance remains inconclusive. Results from this meta-analysis suggest that dietary BG altered the composition of NDF, which promoted the fermentation of soluble fiber in the rumen and resulted in higher microbial protein. In this context, previous research reported that BG can replace a portion of the forage NDF that increases the potentially digestible fraction but reduces DMI in lactating dairy cows (Younker et al., 1998). Moreover, the same study reported that dietary BG decreased DMI but did not affect MY and FE. However, due to the use of animals with both rumen and intestinal cannulas to estimate digestibility and microbial efficiency, such data were not incorporated in the present meta-analysis.

Results from this meta-analysis indicate that BG supplementation did not affect the digestibility of DM, CP, or NDF in dairy cows. These results can be due to the limited number of studies that estimated total-tract digestibility. Conversely, dietary BG decreased DMI, but increased FE across studies. These results could be due to differences in NDF intake, rumen fermentation, and nitrogen efficiency between treatment comparisons. Previous research reported that increasing the level of BG in the diet increased NDF intake, acetate and butyrate proportions in the rumen, and nitrogen intake in feedlot lambs (de Assis et al., 2023). Likewise, dietary BG is a known source of RUP that often promotes intestinal absorption of EAA such as methionine and lysine (Santos et al., 1998). These findings could partially explain why dietary BG tended to increase MY but decreased DMI among treatment comparisons. Furthermore, we hypothesize that increasing the absorption of EAA from BG increased FE as previous research has proposed (Al-Talib et al., 2014).

Collectively, this meta-analysis supports the idea of feeding BG in lactating dairy cows. The dose-response analysis showed that an inclusion level of 20% (∼39% NDF) in the diet can increase MY and FE. However, this meta-analysis failed to establish a clear connection between FE, DMI, and digestibility. Thus, more studies are required to understand the mode of action of BG in the rumen and its effects on the rumen microbiome. Like previous research in small ruminants and dairy cows, higher inclusion levels of BG in the diet (above 30%) can reduce animal performance in ruminants (Erdman and Vandersall, 1983; de Assis et al., 2023). Furthermore, more research is required to investigate the effects of BG on AA metabolism in dairy cows.

This meta-analysis showed that dietary BG increased FE in dairy cows. Dietary inclusion of BG in dairy cows should not exceed 20% to prevent detrimental effects on MY and performance in dairy cattle. Although dietary BG was associated with lower DMI, more research is required to understand its effects on animal performance. Feeding BG to dairy cows requires careful attention to NDF concentration in the diet. Due to the limited data, inconsistent responses were observed in total-tract digestibility. In conclusion, this meta-analysis supports the notion that BG at low concentrations can enhance performance, milk production, and FE in dairy cattle.
